# Insight on Polyunsaturated Fatty Acids in Endometrial Receptivity

**DOI:** 10.3390/biom12010036

**Published:** 2021-12-27

**Authors:** Min Chen, Zimeng Zheng, Jialu Shi, Jun Shao

**Affiliations:** 1 Laboratory for Reproductive Immunology, Hospital of Obstetrics and Gynecology, Shanghai Medical School, Fudan University, Shanghai 200080, China; 20211250016@fudan.edu.cn (M.C.); zzmfdu@126.com (Z.Z.); 21211250041@m.fudan.edu.cn (J.S.); 2 Deparment of Gynecology, Hospital of Obstetrics and Gynecology, Shanghai Medical School, Fudan University, Shanghai 200011, China

**Keywords:** polyunsaturated fatty acids, endometrial receptivity, decidualization, prostaglandins, estrogen, progesterone

## Abstract

Endometrial receptivity plays a crucial role in fertilization as well as pregnancy outcome in patients faced with fertility challenges. The optimization of endometrial receptivity may help with normal implantation of the embryo, and endometrial receptivity may be affected by numerous factors. Recently, the role of lipids in pregnancy has been increasingly recognized. Fatty acids and their metabolites may be involved in all stages of pregnancy and play a role in supporting cell proliferation and development, participating in cell signaling and regulating cell function. Polyunsaturated fatty acids, in particular, are essential fatty acids for the human body that can affect the receptivity of the endometrium through in a variety of methods, such as producing prostaglandins, estrogen and progesterone, among others. Additionally, polyunsaturated fatty acids are also involved in immunity and the regulation of endometrial decidualization. Fatty acids are essential for fetal placental growth and development. The interrelationship of polyunsaturated fatty acids with these substances and how they may affect endometrial receptivity will be reviewed in this article.

## 1. Introduction

Pregnancy is thought to result from the interaction of local secretory factor-mediated embryonic developmental capacity and endometrial receptivity [1]. Therefore, impaired or decreased endometrial receptivity may result in the failure of embryo implantation [2]. Implantation failure is a problem in assisted reproductive technology (ART) that remains unresolved and has raised concerns in the scientific community [3,4]. In Europe, the overall number of ART cycles has continued to increase annually [5]. With scientific and technological advancements, ART has made great progress in terms of embryo selection and cryopreservation technology, but treatment still fails in numerous patients who cannot conceive.

It is well known that the endometrium has a lipid component that is very important for reproduction. Triglycerides and eicosanoids are among the lipid mediators secreted from the endometrium. From the eicosanoid family, prostaglandins (PGs), thromboxanes, leukotrienes, endocannabinoids and sphingolipids have been found to play a role in reproduction [6,7,8]. Fatty acids are divided into three categories, namely monounsaturated, polyunsaturated and saturated fatty acids. Polyunsaturated fatty acids (PUFAs) contain multiple double bonds, and they can be classified into the following three categories according to their double bond positions: n-3, n-6 and n-9 fatty acids. In addition, PUFAs include long-chain unsaturated fatty acids, eicosapentaenoic acid (EPA) and docosahexaenoic acid (DHA) [9,10,11].

These fatty acids play an important role in human growth and development, such as body growth, visual system formation and human reproduction [12]. Linoleic acid is a PUFA commonly found in the daily diet and is derived from vegetable oils such as safflower, sunflower and canola oil [12]. The majority of n-3 unsaturated fatty acids are derived from Alpha [α]-linolenic acid and are found in the chloroplasts of green leafy vegetables. These two important fatty acids can be converted to long-chain unsaturated fatty acids in the liver through desaturation and elongation enzyme systems. In addition to dietary sources, for women of childbearing age, taking PUFA supplements may be beneficial for the treatment of menstrual dysfunction [13]. In animal experiments, researchers found that the addition of n-3 polyunsaturated fatty acids to the feed of heifers improved reproductive efficiency, and the study showed that n-3 polyunsaturated fatty acids had a positive effect on the expression of key genes during the window of implantation [14]. Lipid metabolism is required for uterine receptivity and embryo implantation [15]. The purpose of this review is to describe the potential mechanisms through which PUFAs affect endometrial receptivity.

## 2. Materials and Methods

In order to summarize the relationship of PUFAs and endometrial receptivity, we performed a literature search in PubMed in January 2021. The literature related with PUFAs or endometrial receptivity was included. The search words were “PUFAs” AND “endometrial receptivity” AND/OR “polyunsaturated fatty acids” AND/OR “uterine receptivity” AND/OR “receptive endometrium” AND/OR “endometrium” AND/OR “omega 3”AND/OR “omega 6.” We included studies that clearly or possibly fulfilled the following criteria: PUFAs, endometrial receptivity and reproductive related. In addition, we excluded literature that clearly fulfilled at least one of the following criteria: conference paper, editorial, studies that did not compare the standard measurements with controls and articles written in a language other than English.

Hypothesis: Based on previous research, we found lipid metabolism to be associated with reproductive performance, and some animal research verifies that PUFAs make a difference in their reproduction, but the main mechanisms remain unknown; we assumed that PUFAs play an important role in endometrial receptivity.

## 3. Endometrial Receptivity in Different Mammals

Pregnancy is a multifactorial process that includes embryo implantation, endometrial decidualization, placenta formation and delivery, and it is complex and irreversible [16,17]. Embryo implantation is a complex process involving both the embryo and maternal endometrium and requires the interaction between a embryo and a receptive endometrium to achieve embryo adhesion and embedding in the uterus [18,19]. The ability of the endometrium to allow normal implantation is called receptivity, and optimal receptivity results in a normal implantation process that serves as the basis for a healthy pregnancy [20]. The stage in the endometrial cycle in which the embryo can implant is known as the “window of implantation” [21]. The morphology of the endometrium is characterized by the presence of pinopodes on the luminal epithelium [22]. In addition, the genome of the receptive endometrium suggests the inclusion of multiple response processes, such as an immune response, inflammatory response, an altered complement pathway and coagulation regulation [23]. In all placental mammals, the establishment of contact between the embryo and the mother follows this common critical step, the timing of which varies considerably across species mainly due to the diversity of uterine anatomy and endocrine and molecular interactions [24].

After the formation of a fertilized egg, the single-celled embryo divides into two cells, four cells, eight cells and then becomes a mulberry embryo. After multiple rounds of mitosis, blastocyst formation occurs from differentiated tissues. In most mammals, the blastocyst consists of a layer of trophoblastic ectodermal cells and an inner cell mass (ICM), the former giving rise to the placenta and the latter to the embryo [25]. After the zona pellucida has been stripped, the blastocyst becomes capable of implantation. The duration of the pre-implantation phase of the embryo varies from species to species. In mice, implantation occurs 4 days after intercourse: in humans it is on average 9 days, while in cows, implantation takes place 30 days after fertilization [26]. According to the different types of blastocyst–uterine cell interactions, there are three main types of implantation: central, eccentric and interstitial [27].

The timing of implantation, which corresponds to the beginning of placental development, and the length of gestation in different species are described in Table 1.

## 4. Structure, Synthesis and Classification of PUFAs

**Figure 1:**
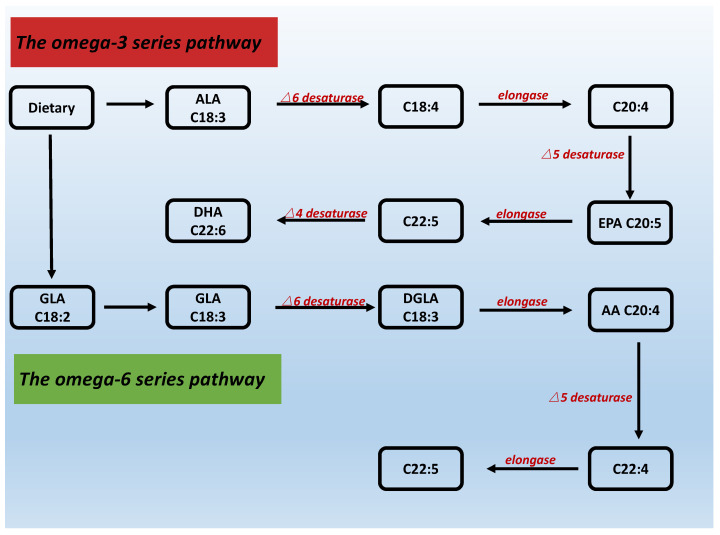
The pathway of polyun-saturated fatty acids n-3 and n-6 series.

Lipids are compounds composed of carbon and hydrogen atoms with a small number of oxygen-containing functional groups.Fatty acids are the main components of membrane lipids and they usually contain 12 to 24 carbon atoms that form hydrocarbon chains. These can be saturated (without double bonds), monounsaturated (with one double bond) and polyunsaturated (with two or more double bonds) fatty acids. Figure 1 presents n-3 and n-6 PUFA series pathways.

Desaturases catalyze the introduction of a double bond between the carboxyl end of the molecule and a pre-existing double bond, introducing further unsaturation into existing PUFAs or synthesizing PUFAs from scratch in mammals. Some animals contain both types of desaturases to produce PUFAs [39]. Front-end desaturases, such as delta-4, delta-5 and delta-6 desaturases, help to introduce a double bond between the carboxyl end of the molecule and the existing double bond, generating PUFAs [40]. Unlike front-end desaturases, methyl-end desaturases, such as delta-12 and delta-15 desaturases, help to add a double bond between the double bond already present in the fatty acid and the methyl end [41]. However, mammals, including humans, do not possess these methyl-terminal desaturases that make essential fatty acids. Therefore, mammals must obtain essential fatty acids, such as linoleic acid (LA) and alpha-linolenic acid (ALA), from food or nutritional supplements (see Figure 1).

## 5. PUFAs, PGs, COX-2, COX-1 and Endometrial Receptivity

There is considerable evidence that the addition of PUFAs to the diet can affect biosynthetic pathways related to PG synthesis and steroid production, and such pathways play several roles in the modulation of reproductive function. The transition into a receptive uterus includes cellular changes in the endometrium and the modulated expression of different cytokines, growth factors, transcription factors and PGs. For example, the PUFA component of the sperm and oocyte membrane is also important for the fertilization process [42]. In human tissues, the synthesis of substances is strictly regulated, as is the synthesis of PGs. Excess PUFAs are often present in the form of esters in cell membrane phospholipids. The first step is to produce multiple PUFA substrates in the cell. The mechanism of action of PUFAs on the production of PGs includes altering the expression of various relevant enzymes and their concentrations, as well as serving as substrates and competitive inhibitors of epoxidation [43]. The ratio of different PUFAs in the diet changes the phospholipid composition of the cell membrane, which is crucial in terms of quantity, since each group of PG precursors is competing for the same enzyme system for metabolism [44]. This, in turn, can have a significant impact on the type of PG synthesis and physiological response [45].

In many species, increased endometrial receptivity involves an increase in vascular permeability at the blastocyst implantation site [46]. It has long been proven that PGs play an important role as vasoactive factors in ovulation, fertilization and late pregnancy leading up to delivery [47]. In addition, PGs have recently been shown to be the key to successful embryo implantation [6,48].

A previous study [49] demonstrated that the addition of n-3 PUFAs in the diet can regulate the gene expression of key enzymes, such as cyclooxygenase (COX) [50], in the PG biosynthesis pathway during early pregnancy, thereby exerting a beneficial effect on the overall reproductive response of sus scrofa. Supplementation with n-3 PUFAs and methionine can improve performance in the second trimester of pregnancy [51].

The central role of PGs in implantation was determined by studies in female mice lacking cytoplasmic phospholipase A2 (cPLA2) or COX-2 enzymes [50]. The lack of these two enzymes results in the loss of PG synthesis, which results in some implantation defects. More specifically, cPLA2 knockout mice demonstrated pregnancy failure and smaller fetal size, both of which are secondary to a delay in implantation. Lysophosphatidic acid receptor-3 (LPA3)-deficient mice exhibit similar issues to cPLA2-deficient mice. However, the administration of exogenous PGs at the appropriate time can restore embryo implantability [6,48].

Prostaglandin E2 (PGE2) maintains the luteal function for embryo development and early implantation. In addition, it induces chemokines expression, which allows trophectoderm to adhere to the decidua for implantation [52]. In a study on pigs, it was found that initiation of progesterone receptors (PRs) in the uterus and uterine epithelium by progesterone 10–12 days after estrus is essential for achieving receptivity of the endometrium for implantation. A review [53] points to the novel role of prostaglandin F2α (PGF2α) in conjunction with PGE2 as embryonic signaling mediators. PGF2α, which was considered undesirable for pregnancy promotion until recently, is now known to stimulate pregnancy-mother interactions and endometrial angiogenesis [54]. Synthesis of large amounts of PGE2 in the endometrium prior to porcine embryo and implantation protects the corpus luteum from lysis [55]. The major progesterone signal estradiol-17β and another factor PGE2 increased endometrial PGE2 secretion by upregulating the expression of PGE2 synthase [53]. Moreover, estradiol-17β has been shown to downregulate the expression of enzymes involved in endometrial PGF2α production [56].

Pathologically, arachidonic acid (AA) is transformed through the COX pathway, triggering the formation of PGs and thromboxane, which is related to fetal growth retardation. The conversion of arachidonic acid to PG is catalyzed by an important rate-limiting enzyme: COX-2. The deletion of COX-2 severely impairs mouse decidualization and embryo implantation [57]. Furthermore, COX-2 can be used to regulate inflammation, differentiation and angiogenesis and can be strongly induced by a variety of stimuli, including cytokines, growth factors and mitogens [58].

It has been also observed that COX-2 can be highly expressed in the endometrium and endometrial stromal cells. Furthermore, previous research [59] demonstrated that COX-2 can inhibit the expression of CD16 in decidual natural killer (dNK) cells and in ectopic endometrial stromal cells. During the implantation window, there is an extensive infiltration and enrichment of nature killer (NK) cells. Another study [60] suggested that COX-2 and its signal transduction pathway-related molecules may play an essential part in the pathogenesis of recurrent spontaneous abortion. COX-2, in reaction to embryonic stimulation (IL-1β), directly results in increased PG biosynthesis in stromal fibroblasts at the implantation site in baboons [61]. The study revealed that COX-2 wild-type embryos can implant successfully in COX-2 deficient mice despite delayed decidualization after implantation [62].

With respect to COX-1, this is also the rate-limiting enzyme that catalyzes the initial step in PG production. Previous research in pregnant pigs found that the mRNA levels of COX-1 increased on days 22–25 (*p* < 0.001), while no upregulation of COX-1 protein expression was detected [63]. Another study found that COX-1 was undetectable between day 8 and day 17 of development in ovine embryos [64]. In rodents, such as mice, a previous study [46] found that, during preimplantation, the COX-1 gene is mainly expressed in the uterine epithelium on day 4 until expression was downregulated in the evening after the onset of the attachment reaction and expressed again in the mesometrial and anti-mesometrial secondary decidual beds on days 7 and 8. These results suggest that PGs produced by COX-1 are involved in decidualization and the persistent endometrial vascular permeability restriction observed during this period [46]. The treatment of ovariectomized ewes with steroids indicated that the expression of COX-1 remained at constant levels regardless of treatment [65]. However, the researchers suggested that the uterine COX-1 gene is influenced by ovarian steroids during early pregnancy [46]. The role of COX-1-derived PGs on day 4 of gestation, when their expression is at the maximum level, is important in the preparation for uterine implantation and COX-2 compensation occurs in the absence of COX-1. COX-1 is associated with increased vascular permeability [66]. Mice with COX-2-inducible subtype null mutations have multifactorial reproductive failure, including ovulation, fertilization, implantation and impaired metaphase function, whereas constitutive enzyme (COX-1)-deficient mice are unaffected in this regard [67].

## 6. PUFAs, Sex Hormones and Endometrial Receptivity

### 6.1. Estrogen

The endometrium is very sensitive to hormone changes, especially in the presence of steroid hormones, and this change prepares the endometrium for embryonic implantation and decidualization [68]. Estrogen and progesterone are critical mediators in embryo implantation and decidualization [36]. Estrogen, in particular, plays an important role during the embryo implantation window, and high levels of estrogen can cause the implantation window to close [69].

It appears that n-3 PUFAs are associated with estradiol. However, two studies investigating the relationship between maternal intake of n-3 and n-6 PUFAs during pregnancy and estradiol levels did not provide definitive results [70,71].

It was found that, in the guinea pig estrous cycle, free PUFA patterns in the plasma may affect steroid hormone concentrations [70]. ALA-rich diets were shown to increase follicular estradiol concentrations in cows, while both LA and ALA decreased progesterone concentrations in the luteal phase [72]. Compared to n-6 PUFAs, n-3 PUFAs were also shown to increase progesterone concentrations in sheep follicles, while estrogen was not affected [68]. These effects are mediated by altering PG synthesis and steroidogenesis, which may be differentially affected by n-3 and n-6 PUFAs [36]. In addition, the effects of these fatty acids play different roles between oestrus and dioestrus. Another study found that DHA stimulated bovine granulosa cell proliferation and steroidogenesis [69]. The positive effect of PUFAs on the rate of sex hormone secretion during the follicular phase may be due to enlarging follicles, thus producing more steroid hormones; overall, it can be hypothesized that PUFAs increased steroid production or altered PG synthesis [73]. Similarly, n-3 long-chain PUFAs were found to affect gene expression as well as estrogen metabolism [74].

### 6.2. Progesterone

Progesterone is an important steroid hormone in the body, and it is also known as the pregnancy hormone due to its important role during that period. During the first week of pregnancy, progesterone is mainly derived from the corpus luteum and, subsequently, as the gestational weeks progress, progesterone is mainly synthesized by the placenta [75]. The mitochondria in the syncytial trophectoderm are the main sites of the synthesis of this steroid hormone, which is synthesized via a two-step reaction [76].

### 6.3. Estrogen and Progesterone Signaling in Endometrial Receptivity

**Figure 2:**
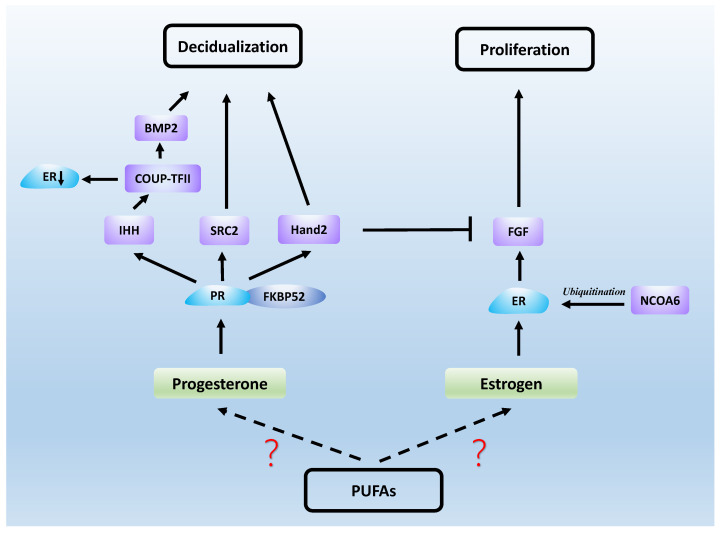
Steroid hormones work with a series of signaling molecules to generate uterine receptivity.

Estrogen and progesterone act mainly through estrogen receptor (ER) and PR, which are both nuclear receptors. It was found that the proliferation of endometrial epithelial cells is mainly achieved by estrogen acting on ERs [77]. ERs in epithelial and stromal cells play an important role in the formation of endometrial receptivity, as well as in epithelial differentiation [78]. FK506-binding protein 52 (FKBP52) is a co-chaperone of PR and, in mice lacking FKBP52, epithelial differentiation fails and uterine receptivity is severely compromised due to decreased progesterone activity and excessive estrogen activity [79]. In addition, knockdown of the nuclear receptor co-activator steroid receptor coactivator2 (SRC2) in the uterus causes implantation failure due to the fact that PR relies on SRC2 to initiate the uterine decidual response [80,81]. Nuclear receptor coactivator-6 (NCOA6) disrupts endometrial receptivity as it degrades ERs through ubiquitination, which results in increased sensitivity of the uterus to estrogen and abnormal expression of progesterone-related genes [82]. Heart and neural crest derivatives-expressed protein 2 (Hand2) is a functional regulator of progesterone and a transcription factor expressed in the uterine stroma. It acts as a repressor of estrogen-mediated epithelial cell proliferation. Fibroblast growth factor (FGF) production maintains epithelial cell proliferation and stimulates the estrogen-induced pathway, and Hand2 expression inhibits this process, which would otherwise result in impaired endometrial receptivity [83]. Another transcription factor, chicken ovalbumin upstream promoter transcription factor-2 (COUP-TFII), is also present in the endometrial stroma and plays an important role in progesterone maintenance of endometrial receptivity. Progesterone first induces the Indian hedgehog (IHH) gene, which regulates COUP-TFII, and bone morphogenetic protein 2 (BMP2) is a downstream molecule induced by COUP-TFII, thereby promoting decidualization [84]. In addition, COUP-TFII causes progesterone inhibition of estrogen by decreasing ERα expression in epithelial cells during the uterine receptive period [85]. Studies on mouse models have also demonstrated that some PR-regulated genes, such as IHH, BMP2 and HAND2, are essential for implantation and decidualization [86]. These results suggest that normal uterine receptivity requires the complexity and accuracy of ER and PR signaling (see Figure 2).

### 6.4. Androgen

Androgen is associated with PUFAs and also plays an important part in endometrial receptivity. Researchers observed that in overweight and obese men, intervention with DHArich fish oil was associated with an increase in testosterone concentrations [87]. Studies have shown that supplementation with n-3 PUFAs has no significant effect on androgen levels in women with polycystic ovarian syndrome(PCOS). However, some pre-term and long-term intervention studies have shown reduced levels of dehydroepiandrosterone (DHEA). Future studies need to be combined with double-blind placebo-controlled clinical trials and long-term follow-up [88]. Polyunsaturated fatty acid diets had better effects on hormone secretion and reproductive parameters in male buffalo compared to saturated fatty acid diets. The addition of n-3 PUFA levels to the ration increased the concentration of testosterone in the plasma and the scrotal circumference of male buffaloes and contributed to a shorter age at puberty [89].

The biological role of DHEA, particularly in fertility, is also controversial. A high androgen level impairs endometrial receptivity in women who have experienced recurrent miscarriages [90]. It was found that testosterone reduced the expression of pinopode and l-selectin ligands in the uterus during receptivity in rats, which may result in the failure of blastocyst implantation under conditions of high levels of this hormone [91]. Testosterone injection results in the loss of intrauterine tight junction complexity and the downregulation of intrauterine claudin-4 and occludin expression during the receptive phase, thus influencing embryo attachment and subsequent implantation [92]. Primary ESC was decidualized in vitro with progesterone and cAMP for 1–8 days with or without the androgen receptor (AR) antagonist flutamide. The addition of flutamide significantly altered the expression of endometrial receptivity indicators. Endometrial androgen biosynthesis during desiccation may play an important role in endometrial tolerance [93]. Another study found that DHEA enhanced in vitro decidualization response to human endometrial stromal fibroblast (hESF) in women of childbearing age. DHEA supplementation during the receptive phase enhances the endometrial function by enhancing the expression of the endometrial receptive marker secreted phosphoprotein 1 (SPP1). Meanwhile, flutamide (an androgen inhibitor) treatment can effectively improve decidualization, angiogenesis and uNK cell production due to hyperandrogenemia, further improving poor endometrial receptivity in PCOS patients [94]. Low concentrations of DHEA were found to increase the antioxidant capacity of metaphase ESCs in mice, and DHEA treatment also improved endometrial tolerance through AR [95]. In conclusion, testosterone is a novel negative regulator of endometrial receptivity.

## 7. PUFAs and Decidualization

In human pregnancy, the embryo first attaches to the luminal epithelium, then invades the endometrial stroma and finally implants in the uterus. This process is influenced by steroid hormones, such as estrogen and progesterone, and the stromal cells that prepare the embryo for implantation undergo significant transformation events. Decidualization consists of morphogenetic, biochemical and vascular changes driven by ERs and PRs. Effective decidualization is a key factor in pregnancy success [96]. The endometrium has to be prepared optimally for a brief period of time before and immediately after the arrival of the blastocyst. PUFAs have been proven to be effective in pregnancy in many animal and clinical studies [97]. We herein summarize the different possible mechanisms through which PUFAs affect the decidualization process.

### 7.1. PUFAs Induce Autophagy in Decidualization by Lipid Peroxidation

**Figure 3:**
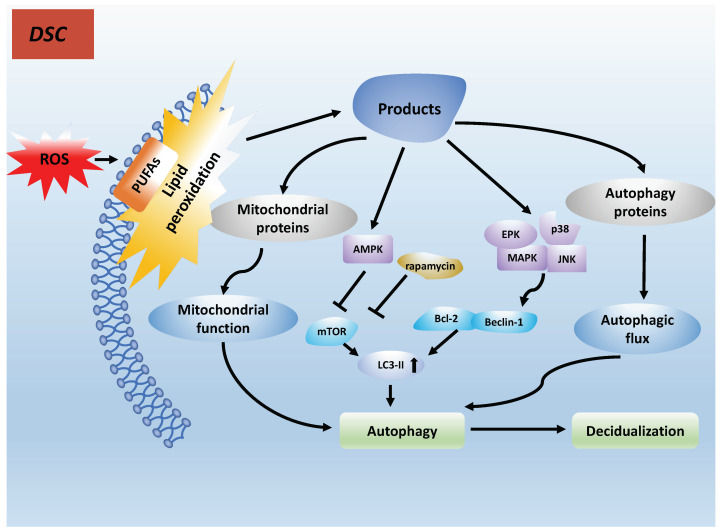
Lipid peroxidation products can adduct specific mitochondrial and autophagy-related proteins, which can result in autophagy and cell death [103].

As cell membranes and organelle membranes contain high levels of PUFAs, they are more susceptible to damage by reactive oxygen species (ROS). This is referred to as lipid peroxidation. Membrane phospholipids possess high levels of PUFAs and are extremely sensitive to ROS attacks [98]. Furthermore, PUFAs can be transformed into reactive free radicals upon their own reaction with free radicals, which can cause a chain reaction of lipid peroxidation. Lipid peroxidation products can adduct to autophagy with specific mitochondrial and autophagy-related proteins, driving cell dysfunction or even resulting in cell death [99]. Lipid peroxidation products trigger autophagic death via three different signaling pathways, such as the AMPK/mTORC pathway [100], the mTOR pathway [101] and the JNK-Bcl-2/Beclin 1 pathway [102] (see Figure 3).

Rapamycin, a major sensitive inhibitor of mTORC, increases LC3-II levels through its inhibitory effect on mTORC1 signaling and, thus, results in cellular autophagy [104]. In addition, n-3 PUFAs activate the Galphaq-p38/MAPK signaling pathway, resulting in autophagy in breast cancer cells [105] and macrophages [103]. As is discussed above, defective decidualization is considered to be a key factor resulting in compromised endometrial receptivity and implantation failure. Autophagy is an important step in the decidualization process [106]. Mestre Citrinovitz et al. [107] found that the decline in ATP levels during the decay of endometrial stromal cells (ESCs) was accompanied by an increase in autophagy flux. In addition, they knocked out ATG7 and ATG5, establishing an autophagy-deficient cell model, and found that the decidualization process was impaired, confirming the role of autophagy in the normal decidualization process [108].

### 7.2. PUFAs Acting on the Receptor GPR120 Result in Decidualization

Receptor GPR120 is a member of the G protein-coupled receptor erythrocyte family, which mediates powerful anti-inflammatory and insulin sensitization effects [109]. GPR120 serves as a receptor for PUFAs [110]. Huang et al. showed that GPR120 promotes decidualization through the upregulation of Forkhead box-O1 (FOXO1) and glucose transporter-1 (GLUT1) expression, glucose uptake and activation of the pentose phosphate pathway in endometrial stromal cells [111].

### 7.3. PUFAs, COX-2 and Decidualization

EGFR signaling, COX-2 expression and PG signaling play important roles in the decidualization of ESCs, and these processes are regulated through the lipid mediator lysophosphatidic acid (LPA) [112]. COX-2 can activate peroxisome proliferator-activated receptor-δ (PPAR-δ) and retinoic acid X receptor (RXR), which are known as important regulators of decidualization and embryo implantation [113]. A previous study [114] suggested that both PGE2 and PGI2 are derived from COX-2 and mediate their functions through EP2 and PPAR-δ receptors during the early stages of decidualization in mice. In addition, PUFAs can affect these transcription factors as endogenous ligands and PPARs are a family of nuclear receptors [115].

In prior studies, the treatment of decidual cells with n-3 unsaturated fatty acids reduced the production of uterine PGs, because decidual cells may play a key role in the production of PGE2 and PGF2α in the uterus [116]. In addition, n-6 PUFAs are associated with an increased production of PGs in decidual cells. n-6 PUFAs can stimulate PG production, which was consistent with in vitro and in vivo observations; in humans and rats, dietary supplementation with n-6 PUFAs is associated with a shorter gestation [117].

## 8. LPA and S1P Can Influence the Endometrial Receptivity

Glycerophospholipids are the part of lipids, which are synthesized by glycerin, fatty acids (including PUFAs), choline, serine, etc. PLA1 catalyzes the hydrolysis of the sn-1 site of glycerophospholipids to produce fatty acids and lysophospholipids, while PLA2 catalyzes the sn-2 site to produce fatty acids and lysophospholipids [118]. LPA and sphingosine 1-phosphate (S1P) are representative lysophospholipids, and they both play important roles in reproductive processes.

### 8.1. LPA

The LPA3 signaling pathway plays an established role in the regulation of male and female reproduction, and its dysregulation is associated with mammalian sterility and a number of reproductive problems [119]. Indeed, LPA3−/− mice exhibit delayed embryo implantation, embryo crowding and reduced litter size. These defects were traced to maternal effects, as wild-type mouse embryos failed to implant correctly after transfer to LPAR3−/− maternal lines [6]. Mice lacking the COX-2 enzyme (an enzyme that produces PGs and is located downstream of LPA3) have a similar defective phenotype. This suggests that the exogenous addition of PGs can resolve the problem of delayed implantation and reduced litter size, which also suggests that the LPA3-mediated signaling pathway is upstream of PG synthesis [6]. However, the LPA3 signaling pathway mediates implantation in a PG-dependent and non-dependent manner; therefore, this treatment failed to reverse embryo crowding [120]. Furthermore, LPA is also related to decidualization, and a previous study uncovered a novel mechanism of LPA3-mediated decidualization during implantation; their results indicate that ATX/LPA/LPA3 signaling induces decidualization through classical heparin-binding EGF-like growth factor (HB-EGF) and COX-2 pathways [117].

### 8.2. SIP

The sphingolipid metabolic pathway produces biologically active signaling metabolites and complex lipids for membrane organization and structure. A prominent signaling lipid is S1P, which promotes cell survival as well as proliferation [121,122]. Sphingosine kinase (Sphk) is an essential enzyme in the sphingolipid metabolic pathway, and two isoforms of mammalian Sphk are known (Sphk1 and Sphk2) [123]. The study found that SPHK−/−SPHK−/+ mice are infertile because of impaired decidualization [8]. In studies with sheep, the expression of S1P-related enzymes and S1P receptors in the endometrium was found to be spatiotemporally specific, and one of the S1P synthases, SPHK1, was associated with maternal–fetal interface angiogenesis, providing blood support for sheep pregnancy [124]. In human and mouse experiments, the key enzymes for S1P synthesis and degradation were found to be elevated during pregnancy, as well as S1P receptors [125,126]. The expression of COX-2 can be upregulated by S1P in DSCs [126]. It has been demonstrated that, during normal pregnancy, the sphingolipid metabolic pathway is highly activated during the decidualization process. Disruption of the activation pathway by the Sphk gene alters sphingolipid metabolite levels, resulting in metaphase defects and early pregnancy loss [8].

## 9. PUFAs in Immune Regulation

### 9.1. Immune Cells

As mentioned above, PUFAs include n-3 and n-6 fatty acids. These PUFAs can synthesize PG and leukotrienes, increase the permeability of blood vessels, induce lysozyme release, promote chemotaxis of white blood cells and serve a physiological role in immune response. A previous review [127] reported that a PUFA-enriched diet can stimulate the phagocytic ability of macrophages, promote NK cell response and enhance non-specific immune function. In addition, different doses of PUFAs can regulate different aspects of the body’s specific immune function. PUFAs, as a response factor of cytokine gene transcription in the body, can affect signal transduction and gene expression, thereby affecting the function of immune cells. Endometrial biopsies exhibited an increased expression of different pro-inflammatory cytokines and an abundance of macrophages and dendritic cells (DCs) [128]. Feeding experimental animals with fish oils that provide EPA and DHA result in higher levels of PUFAs in lymphocytes [129], macrophages [130], Kupffer cells [131] and neutrophils [132,133].

Macrophages play an indispensable role in the innate human immune system. These cells accumulate in the gestational uterus prior to embryo implantation and remain in the decidual cells during pregnancy [134,135]. N-3 fatty acids cause important changes in macrophage gene regulation. Adding PUFAs, such as DHA and EPA, to cultured macrophages was shown to change their miRNA expression profiles. This may be the mechanism through which n-3 fatty acids reduce inflammatory responses [136,137]. Decidual macrophages become important regulators of dNK cells by secreting IL-15, which induces the differentiation of resting NK cells into activated dNK cells in the endometrium, thus acting as a promoter of decidualization [138].

It is well known that dendritic cells (DCs) have a crucial function in the adaptive immune system [139,140]. The MHC II and costimulatory molecules on the surface of DCs can be downregulated by n-3 fatty acids [141]. The result is that the activation of T cells by DCs exposed to n-3 fatty acids is severely impaired. It was previously suggested that the exhaustion of E2 can result in the severe impairment of embryo implantation and embryonic resorption [142]. Nevertheless, the effects observed may have been associated with chemotactic success instead of immune tolerance. Another study showed that, in a mouse model, DC treatment dramatically reduced the spontaneous uptake rate [143]. The DCs of the uterus are very important in the pregnancy, especially in regard to their involvement in the maternal-fetal immune response. The results demonstrated that endometrial tissue remodeling and growth can be controlled by Th1/Th2 cytokines, which can be secreted by uterine dendritic cells (uDCs) [144,145]. DCs and macrophages create an inflammatory gradient that affects the formation of a mucin layer by epithelial cells and increases the expression of blastocyst adhesion molecule ligands. This inflammatory gradient allows the attachment and adhesion of blastocysts to epithelial cells and promotes implantation, thereby increasing endometrial receptivity [146].

In the first trimester of pregnancy, T cells make up 10% to 20% of immune cells [147]. Studies from several in vitro and animal models have shown that omega-3 PUFAs in the diet generally exert a suppressive effect on T-cell function [148,149]. In fact, n-3 fatty acids are beneficial for certain T-cell-mediated diseases [150]. However, no difference was found in the percentage of T cells in the blood of rats receiving a diet high in n-3 PUFAs [151], although feeding n-3 PUFAs to mice with experimentally induced colitis can affect the T cells, such as regulatory T cells (Tregs), Th22, Th1, Th2 and Th17 cells [151]. Nevertheless, when n-3 PUFAs were administered to human subjects, the researchers observed no change in the percentage of T cells or T-cell populations in the blood [152,153]. Tregs regulate other leukocytes to influence decidual response, and Tregs are also essential for the regulation of maternal immune tolerance [99].

Inadequate decidualization is widely recognized as one of the major causes of spontaneous abortion, and there is a large enrichment of NK cells present in the decidua. The evidence on whether PUFAs can affect NK cell function is insufficient and, often, contradictory. It has been reported that dietary DHA stimulates the activation of splenic NK cells in mice [154]; however, in influenza-infected mice, the opposite result was observed [155]. Increasing dietary EPA did not change the number of circulating NK cells in the blood [152]. However, another similar study found that providing DHA and EPA in the diet can decrease the percentage of NK cells [153]. Appropriate doses of EPA, but not other n-6 or n-3 PUFAs, may reduce NK cell activity in healthy subjects [156].

### 9.2. Inflammation and Cytokines

Considering that embryo attachment and endometrial invasion require a connection with the maternal vascular system, the implantation process may be considered as a pro-inflammatory response [157]. Recent pregnancy studies have shown that n-3 PUFAs affect not only the maternal but also the fetal immune system [158,159,160], which can reduce the risk of developing of allergic diseases in the neonate [159] and also later in life [161]. In a study of patients undergoing in vitro fertilization, the levels of cytokines and immune cells produced during pregnancy were found to be positively correlated with pregnancy outcomes [128]. It was found that the role of cytokines, such as IL-6, CX3CL1 and IP-10, includes not only attracting and activating immune cells but also participating in the implantation process via human trophoblast cells [162,163,164]. In a trial for psoriasis drug development, researchers administered an agent containing n-3 PUFAs to the patients and found that their NK cells were suppressed, and cytokine production was reduced, resulting in a lower immune response [165]. Diets rich in EPA or DHA can reduce the expression of IL-2 receptor chain mRNA in T cells, which results in lower IL-2 secretion. It is well known that cytokine levels are increased during early implantation [166,167]. Endometrial cells and immune cells secrete these cytokines, which are usually enriched at the site of embryonic implantation, playing an important role in endometrial receptivity [168].

### 9.3. Adhesion Molecules

Researchers found that EPA is the omega-3 fatty acid responsible for the improvement of the formation of cytokines and the reduction in the induction of pro-inflammatory adhesion molecules (selectin, intercellular adhesion molecule-1 (ICAM-1) [169]). Another study also found that DHA, reduced the expression of adhesion molecules on the surface of cultured endothelial and monocytes, such as vascular cell adhesion molecule-1 (VCAM-1) and ICAM-1 expression [170]. Selectins belong to the cell adhesion molecule (CAM) family, which includes p-selectin, l-selectin and e-selectin. As for humans, l-selectin plays an important role in the process of implantation [171]. The selectin adhesion system was well established at the maternal–fetal interface. On the blastoderm side, intense l-selectin staining was observed over the entire embryonic surface. On the maternal side, the expression of selectin oligosaccharide ligands was upregulated during the window of embryo attachment [172].

## 10. Hypothalamic-Pituitary-Adrenal(HPA)axis, PUFAs and Endometrial Receptivity

Any intrinsic or extrinsic stimulus that elicits a biological response is referred to as stress. It is a real or perceived state of threat to health of the organism [173]. The HPA axis, as part of the physiological adaptation to stress, mediates the function of the hypothalamic-pituitarygonadal (HPG) axis, which is responsible for the maturation of the reproductive organs and the reproductive capacity of the body. The HPG axis controls the reproductive system by using endocrine signals from gonadotropin-releasing hormone (GnRH), which is secreted by the hypothalamus [174]. Female mice with an increased level of glucocorticoids had marked apoptosis of oviductal cells, poor embryonic development and a reduced number of blastocysts [175]. Under stress, the pregnancy rate and mean litter size of pregnant rats were significantly smaller than those of controls, partly due to a reduction in the implantation site of the rat’s endometrium [176]. The results showed that female mice subjected to inhibitory stress had reduced endometrial cell proliferation, vascular endothelial growth factor expression and microvascular density, which supports the findings of the previous study [177]. It has been reported that auditory stress in female mice also adversely affects the receptivity of the uterus [178]. It was found that combined exposure to heat and psychological stress lresulted in disruption of the estrous cycle in mice, as evidenced by significantly prolonged estrus, reduced endometrial tolerance biomarkers and impaired uterine structures [179]. These results suggest that different types of stress input can negatively affect endometrial tolerance. In addition, the researchers found that n-3 PUFA improved HPA axis abnormalities [180]. We hypothesized that n-3 PUFAs could improve endometrial receptivity in this manner. This study also supports our hypothesis that the strongest evidence for the role of dietary interventions in depression is that the use of high doses of EPA in patients with depression improves the symptoms of depression; thus, it can be used as an adjunctive medication [179].

## 11. PUFAs and IVF Treatment

Several prospective cohort studies have examined the relationship between serum PUFAs and in vitro fertilization outcome measures and success, but the results have been inconsistent. A study investigated approximately 200 women and recorded their diet for 4 weeks prior to in vitro fertilization, specifically the intake of n-3 PUFAs in their diet. It was found that the estradiol concentrations in the body increased with high intake of ALA, while an increased intake of EPA and DHA could result in lower follicle numbers and altered embryo morphology; however, the study sample was too small and the clinical effects of PUFAs must be further investigated [181]. Jungheim et al. [182] found that, in women undergoing in vitro fertilization, higher ALA levels resulted in lower pregnancy rates, and serum ALA levels did not significantly correlate with estradiol levels or oocyte quality; moreover, ALA levels were weakly negatively correlated with embryo implantation. Whether this effect is associated with an excessive intake of ALA is unclear. In other studies, no single PUFA was found to have a correlation with pregnancy success, but women with high linoleic acid-α ratios had a higher chance of becoming pregnant than women with low linoleic acid-α ratios [183].

Another small study found that n-3 PUFA intake did not affect in vitro fertilization outcomes [184]. By measuring the serum and follicular fluid fatty acid levels in 105 women undergoing in vitro fertilization, it was found that the level of EPA in the body is associated with pregnancy, as EPA is significantly higher in pregnant women; in addition, total fatty acid levels were associated with the quality of the follicles [185]. In addition, couples who consumed diets with high fish oil content had higher conception rates [186]. However, the role of these PUFAs in early pregnancy is unclear [43]. Several animal studies have indicated that increased dietary PUFA content has a direct effect on oocyte quality and embryo quality, whereas other effects include steroid formation (described above) and embryo implantation in the uterus, although the exact mechanisms must be further investigated [187].

## 12. Conclusions and Prospect

Preimplantation embryonic development and uterine preparation for implantation are two major factors affecting female fertility. The main method currently used to predict endometrial receptivity is a transvaginal ultrasound to assess endometrial thickness, volume and perfusion. The success rate of embryo transfer when using ultrasound and hormone levels to assess uterine receptivity is 50%. Therefore, there is a need to identify biomarkers for assessing endometrial receptivity and selecting the right time for embryo transfer. PUFAs play an important role in the body by producing PGs, estrogen and progesterone, which are related to the receptivity of the endometrium. However, their main mechanisms of action have yet to be fully elucidated.

In summary, PUFAs can affect several reproductive processes. It is widely believed that fertility rates are currently declining in both humans and farmed animals. PUFAs appear to be a double-edged sword, as some are necessary, but excessive amounts may be harmful. To date, the answer to the question of how to achieve an optimal fertility balance at different stages of life remains largely unknown.

## Figures and Tables

**Table 1:** The timing of the implantation, which corresponds to the beginning of placental development and the length of gestation between species.

Species	Development to Blastocyst	Apposition and Adhesion	Implantation	Type of Implantation	Relevant References
Human	Day 6	Day 7/8	Day 8	Interstitial	[26]
Mouse	Day 4	Day 4	Day 4–5	Eccentric	[28]
Cow	Day 7	Days 19–22	Day 30	Centric	[29]
Pig	Day 6	Days 16–18	Days 20–22	Centric	[30,31]
Domestic cat	Day 7	Day 12	Day 14	Centric	[32]
Domestic dog	Day 7	Day 20	Day 20	Centric	[33,34]
Cheetah	Day 7	Day 19	Day 21	Centric	[35]
Rats	Day 5.5	delay implantation	delayed implantation	eccentric	[36]
Hamsters	Day 3	Day 4	Day 4	eccentric	[37]
Guinea pigs	Day 6	Day 6/7	Day 7	Interstitial	[38]

## Data Availability

Not applicable.

## Abbreviations

The following abbreviations are used in this manuscript:

ART	Assisted reproductive technology;
AA	Arachidonic acid;
ALA	Alpha-linolenic acid;
AR	Androgen receptor;
BMP2	Bone morphogenetic protein 2;
CAM	Cell adhesion molecule;
COUP-TFII	Chicken ovalbumin upstream promoter transcription factor-2;
COX	Cyclooxygenase;
cPLA2	cytoplasmic phospholipase A2;
DCs	Dendritic cells;
DHA	Docosahexaenoic acid;
DHEA	Dehydroepiandrosterone;
dNK	Decidual natural killer;
DSCs	Decidual stromal cells;
EPA	Eicosapentaenoic acid;
ER	Estrogen receptor;
ESCs	Endometrial stromal cells;
FKBP52	FK506-binding protein;
FOXO1	Forkhead box-O1;
GLUT1	Glucose transporter-1;
GnRH	Gonadotropin-releasing hormone;
Hand2	Heart and neural crest derivatives-expressed protein 2;
HB-EGF	Heparin-binding EGF-like growth factor;
HPA	Hypothalamic-pituitary-adrenal;
HPG	Hypothalamic-pituitary-gonadal;
ICAM-1	Intercellular adhesion molecule-1;
ICM	Inner cell mass;
IHH	Indian hedgehog;
LA	Linoleic acid;
LPA	Lysophosphatidic acid;
LPA3	Lysophosphatidic acid receptor-3;
NCOA6	Nuclear receptor coactivator-6;
NK	Natural Killer;
PCOS	Polycystic ovarian syndrome;
PGE2	Prostaglandin E2;
PGF2α	Prostaglandin F2α;
PGs	Prostaglandins;
PPAR-δ	Peroxisome proliferator-activated receptor-δ;
PPARα	peroxisome-activated receptor-alpha;
PRs	Progesterone receptors;
ROS	Reactive oxygen species;
RXR	Retinoic acid X receptor;
S1P	Sphingosine 1-phosphate;
Sphk	Sphingosine kinase;
SPP1	Secreted phosphoprotein 1;
SRC2	Steroid receptor coactivator2;
Tregs	Regulatory T cells;
uDCs	uterine Dendritic cells;
VCAM-1	Vascular cell adhesion molecule-1.
